# Emergence of the fungal immune system

**DOI:** 10.1016/j.isci.2023.106793

**Published:** 2023-05-02

**Authors:** Asen Daskalov

**Affiliations:** 1 State Key Laboratory for Managing Biotic and Chemical Treats to the Quality and Safety of Agro-products, Institute of Plant Protection and Microbiology, Zhejiang Academy of Agricultural Sciences, Hangzhou, China; 2 ImmunoConcEpT, CNRS UMR 5164, University of Bordeaux, Bordeaux, France

**Keywords:** Immunology, Microbiology, Cell biology

## Abstract

Investigation of fungal biology has been frequently motivated by the fact that many fungal species are important plant and animal pathogens. Such efforts have contributed significantly toward our understanding of fungal pathogenic lifestyles (virulence factors and strategies) and the interplay with host immune systems. In parallel, work on fungal allorecognition systems leading to the characterization of fungal regulated cell death determinants and pathways, has been instrumental for the emergent concept of fungal immunity. The uncovered evolutionary *trans*-kingdom parallels between fungal regulated cell death pathways and innate immune systems incite us to reflect further on the concept of a fungal immune system. Here, I briefly review key findings that have shaped the fungal immunity paradigm, providing a perspective on what I consider its most glaring knowledge gaps. Undertaking to fill such gaps would establish firmly the fungal immune system inside the broader field of comparative immunology.

## Graphical abstract

**Figure:**
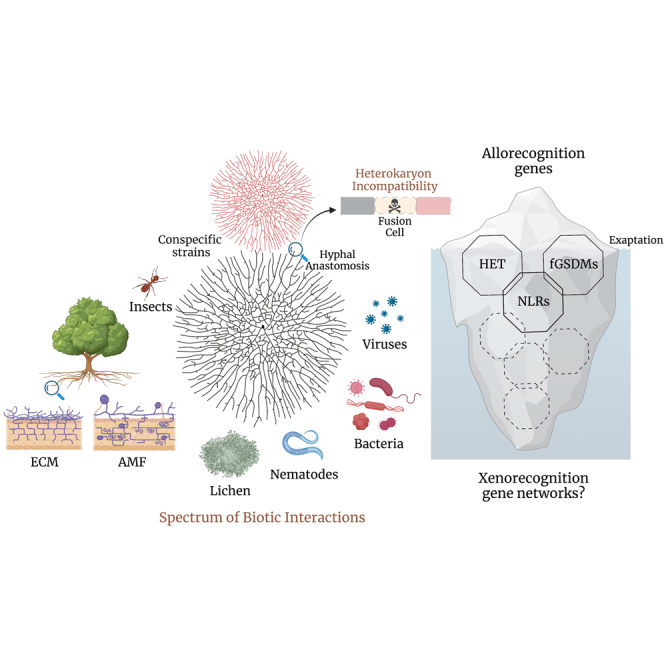

## Introduction

The contemporary view of the immune system fully integrates its many physiological roles (i.e., reproduction, embryogenesis, development etc.), which elevates it to a system ensuring organismal integrity, homeostasis and the mediation of a broad range of biotic interactions.^1^^,^^2^^,^^3^ Despite this recent conceptual evolution, the historically predominant view of the immune system as a machinery dedicated to primary fend off pathogens persists, highlighting one of its best-characterized and essential roles. Defense from infections often relies as a last resort on programs of cellular suicide; a variety of regulated cell death (RCD) reactions eliminating infected host cells to prevent pathogenic spread and induce downstream response.^4^^,^^5^^,^^6^ Germline-encoded molecular pathways of immune-related cell death have been extensively studied in vertebrates with emphasis on mammals (humans, mice)^7^^,^^8^ and invertebrates such as insects.^9^^,^^10^ Innate immune systems have also been described outside of animals – in plants,^11^ bacteria^12^ and archaea.^13^ In these phyla also, RCD represents a key defense strategy coined *hypersensitive response*^14^ and abortive infection (Abi),^15^ in plants and bacteria respectively. Notwithstanding the extreme phylogenetic distances between these taxa, some of their characterized defense pathways share evolutionary ties, which may concern molecular receptors, signaling accessory domains or cell death execution proteins. These findings suggest that organismal defense pathways in different branches of the Tree of Life can share a common, ancient evolutionary origin.

In fungi, one of the main eukaryotic kingdoms with millions of species,^16^ the concept of an immune system is still in its infancy and underexplored, as exemplified by the handful of publications approaching the topic directly.^17^^,^^18^^,^^19^ The notion of fungal immunity has however appeared in a variety of research contexts, including studies on mycoviruses,^20^ organismal defense against other microbes and predation,^21^^,^^22^^,^^23^ fungal-bacterial interactions^24^^,^^25^ and the non-self discrimination phenomena occurring between conspecific fungal strains, known as allorecognition.^26^^,^^27^^,^^28^ It is decidedly from the latter that a compelling paradigm for a fungal immune system has progressively taken shape over the last decade, supported by discoveries drawing evolutionary parallels between fungal RCD pathways and eukaryotic immune systems. The established parallels allow us to situate the topic in the broader context of comparative immunology, and my perspective here focuses mainly on that point. Moreover, I discuss some of the knowledge gaps, which need to be addressed for the establishment of additional functional and conceptual analogies between the fungal immune-related cell death pathways and other immune systems. Systematizing recent findings on fungal non-self recognition programs in an immunological framework might represent a valuable contribution to the field of comparative immunology and offers a promising outlook for the studies of regulated cell death in the vast, ecologically and economically important clade of fungi.

## Fungal allorecognition – ‘Tip of the iceberg’ of the fungal immune system

Our understanding of the fungal immune system remains poor. However, significant progress has been made in establishing a paradigm of fungal immunity since Paoletti and Saupe, more than a decade ago, suggested that regulated cell death occurring during fungal conspecific non-self recognition (allorecognition), has its origins in cell death pathways dedicated to heterospecific non-self recognition (xenorecognition).^19^ The Paoletti-Saupe hypothesis offered a novel and intriguing interpretation regarding the evolutionary origins of fungal allorecognition, which has been studied for almost a century,^29^ and initiated the integration of the phenomenon into the field of comparative immunology.

Allorecognition – defined as the ability of an organism to discriminate its biological self from non-self of the same species – is a widespread phenomenon initially described in the context of graft rejection^30^ and naturally occurring in mammals, colonial marine invertebrates and protochordates,^31^^,^^32^^,^^33^ slime molds,^34^^,^^35^ bacteria,^36^ and fungi.^37^^,^^38^^,^^39^ Fungal allorecognition can occur at distance (pre-contact),^40^ at the contact point between different strains^41^ – blocking cellular fusion (*anastomosis*) – or post-cellular fusion.^42^ When anastomosis occurs during the sexual cycle between gametes of different strains, the allorecognition process can result in sexual incompatibility.^43^ Alternatively, anastomosis between somatic cells (*hyphae*) often leads to vegetative (or heterokaryon) incompatibility.^28^^,^^37^^,^^44^ The heterokaryotic fusion cells undergo RCD. The process is characterized by microscopic hallmarks such as increased and abnormal septation, lipid droplets accumulation, hyper-vacuolization and culminating in cellular lysis, reestablishing strict colonial individuality.^45^^,^^46^^,^^47^ Recent work has established that a single locus can control both sexual and vegetative incompatibility in the genus *Podospora*, playing a role in speciation.^48^

Vegetative incompatibility (VI) prevents cytoplasmic mixing between genetically incompatible strains, contributing toward the preservation of organismal individuality (one genome - one organism). The allorecognition reaction thus acts akin to a defense system limiting resource plundering by aggressive genotypes^49^ and the spread of cytoplasmically transmitted fungal viruses (*mycoviruses*).^50^^,^^51^ Mycoviruses are frequently found in fungal species and have occasionally been shown to reduce host growth and impact negatively the virulence of pathogenic species, reducing their fitness.^52^^,^^53^ VI-dependent mycoviral protection has been best characterized in the plant pathogen *Cryphonectria parasitica*, causative agent of the chestnut blight disease and a model organism for the study of vegetative incompatibility and virus/host interactions.^54^^,^^55^ In *C. parasitica*, five of the six identified allorecognition systems limit horizontal mycoviral transmission.^56^ Anti-viral effects of vegetative incompatibility have been reported in several other model fungal species, belonging to *Neurospora*,^51^*Podospora*^57^ and *Aspergillus* genera.^50^^,^^58^ In line with this prophylactic anti-viral role of VI, it was found that mycoviral infection could suppress regulated cell death by incompatibility.^59^^,^^60^

Allorecognition genes, termed *het* (heterokaryon) or *vic* (*v*egetative incompatibility), are bi- or multiallelic and highly polymorphic with allorecognition systems comprising often two tightly linked genes.^37^ When characterized at molecular level, *het* alleles tend to be distributed evenly in a population, exhibiting marks of balancing selection.^39^^,^^48^^,^^61^^,^^62^ Balanced allelic distribution likely results from frequency-dependent selection, where rare alleles confer vegetative incompatibility with higher number of strains compared to more common alleles. Strains carrying rare alleles would undergo higher percentage of abortive anastomosis with other conspecific individuals and thus benefit from an extended protection. In time, *het* alleles frequencies would tend to equilibrium, favoring the maintenance of high intraspecies genetic diversity. Observed evolutionary signatures on characterized *het* genes represent thus indirect evidence for the fitness advantages procured by allorecognition, including as a prophylactic anti-viral defense mechanism. As may be expected, the upper limit of *het* loci that can be maintained in a given species is reached when emergence of another incompatibility system does not provide further fitness advantage in terms of protection against mycoviral or other parasitic genetic elements, because of the functional redundancy between *het* systems.^63^ Hence, allorecognition loci are usually ranging between 8 and 12 per species, a number high enough for the generation of hundreds of distinct vegetative compatibility groups (VCGs), resulting in abortive fusions for the vast majority of pairwise conspecific interactions. The numbers of observed unlinked *het* loci are thus near the theoretical maximums.^64^

An alternative hypothesis aiming to explain the diversifying selection observed on some *het* genes,^65^ introduced by Paoletti and Saupe,^19^ proposes that genetic novelty is regularly selected for in an arms race between fungi and their pathogens. The pathogens could be mycoviruses, bacteria^66^^,^^67^ or amebae.^68^ In this case, the genesis of allorecognition systems is likely a by-product of a continuous organismal co-evolution, following Van Valen’s Red Queen evolutionary principle,^69^ between fungal hosts and their biotic antagonists. Consequently, some RCD pathways, evolving to track and prevent the spread of pathogens, have been co-opted through exaptation^70^ for the purposes of regulating conspecific anastomosis. The Paoletti-Saupe hypothesis puts the emphasis on xenorecognition as the driver behind *het* genes diversification but is non-exclusive of the balancing selection operating on allorecognition systems. Thus, some genes dedicated to non-self discrimination are likely subjected to evolutionary pressure from simultaneously allo- and xenorecognition processes. Such dynamics could explain that the observed polymorphism on some *het* genes greatly exceeds the number of incompatibilities those genes define.^71^ Furthermore, the ties between allo- and xenorecognition could explain why the cellular manifestations of VI are highly pleiotropic with a massive transcriptional reprogramming, induction of autophagy, induction of secondary metabolism clusters, induction of numerous toxins, and cell wall thickening. The transcription response appears overwhelming and unlikely to have been specifically tailored for the elimination of fusion cells between conspecific fungal strains. In terms of its cellular manifestations, the incompatibility reaction shows some analogies to pathogens defense responses observed in other organisms.^72^

**Figure 1:**
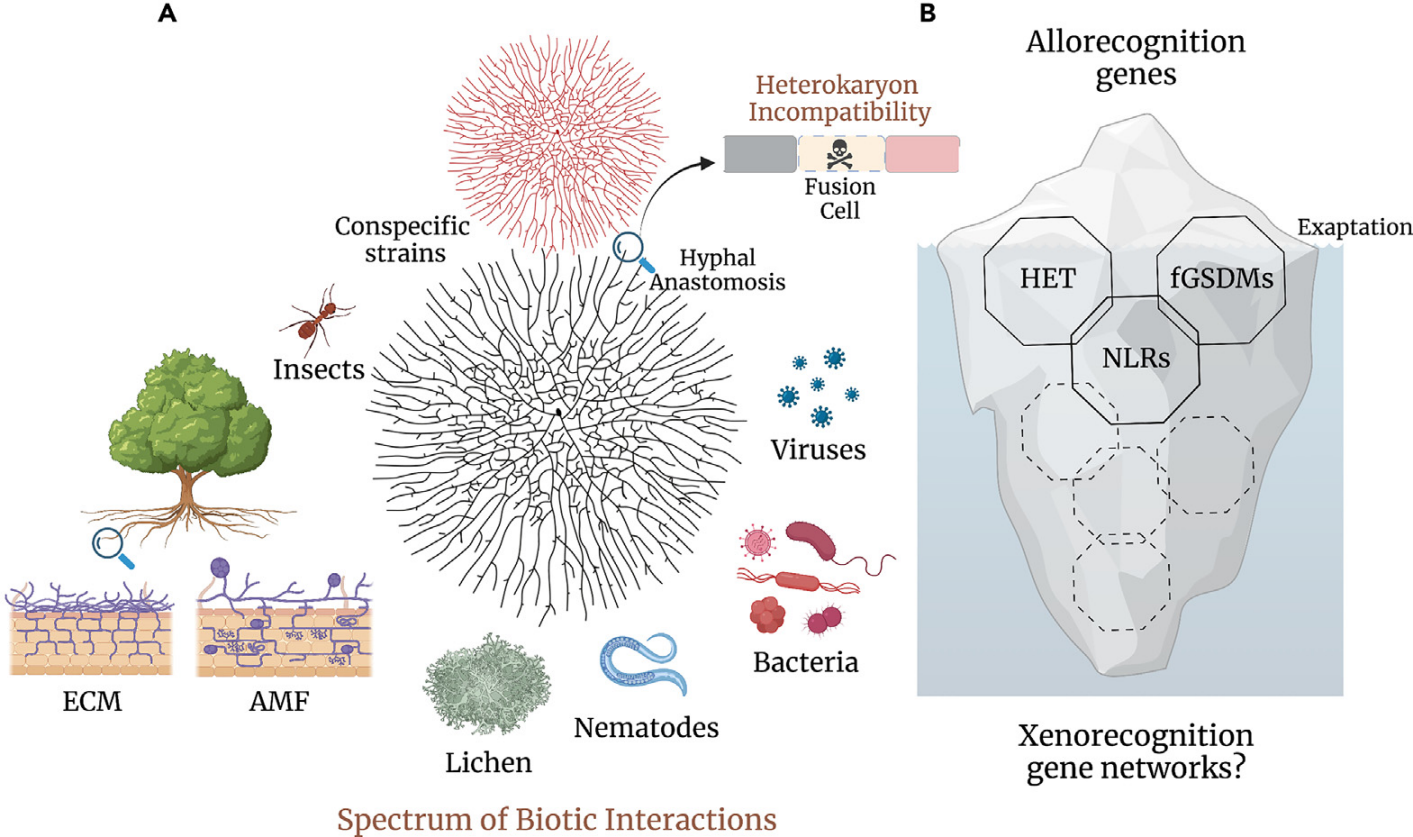
Allo- and xenorecognition in fungi: two sides of the same coin (A) Fungi exhibit a great diversity of lifestyle and are involved in a broad range of biotic interactions. These can involve different levels of heterospecific antagonism or various forms of symbiosis (xenorecognition) or interactions with other fungal individuals from the same species (allorecognition). Frequently, hyphal fusions between genetically distinct conspecific individuals – illustrated by the red and black mycelial colonies above – would be abortive and result in the elimination of the heterokaryotic fusion cells through a regulated cell death reaction. (B) Symbolic representation of the fungal immune system as a cartoon of an iceberg. The iceberg model represents the allorecognition genes as the tip of the iceberg (fungal lifestyle specific defense) and the immune-related gene networks as the submerged side of the iceberg (xenorecognition networks). The model reflects simultaneously the relative sizes of the two gene pools, the accumulated experimental knowledge and the relations between these two sides of the fungal immune system. As allorecognition has an adaptive value and represents an organismal defense reaction against conspecific non-self, the genes that have been recruited in the process acquire a new function, although in the same biological context (immunity) and thus have been the object of an evolutionary exaptation. Allorecognition genes have been most often exaptated from three gene families (solid line octagons): HET-domain encoding genes, NOD-like receptors (NLRs) genes and gasdermin (fGSDMs) encoding genes. Dashed line octagons indicate that other immune-related gene families will likely be identified in the future.

Paoletti and Saupe bring forward other key arguments to support their hypothesis. Although allorecognition systems are usually limited in number (ranging in the 8–12 per species), characterized *het* genes very often belong to much broader gene families,^45^^,^^73^ comprising dozens or hundreds of genes per genome. Moreover, one of the gene families encodes for members of a *trans*-kingdom group of immune receptors.^74^ Considering these two remarkable findings, one could foresee that several gene families and networks, dedicated to heterospecific non-self recognition, provide also a reservoir for the recruitment of novel *het* genes and the emergence of novel allorecognition systems. Thus, a simplified model integrating both aspects of organismal defense in fungi (allo- and xenorecognition) could be represented as a conceptual iceberg with a visible side, corresponding to the historically characterized allorecognition cell death and the genes controlling it, and a submerged, hidden side, corresponding to broader immune networks and pathways, from which allorecognition systems emerge (Figure 1). The iceberg representation of the allo-/xenorecognition genetic relations in fungi captures the core idea of the Paoletti-Saupe hypothesis and despite being a simplistic model, provides a visual hint toward the direction of future research work.

Although our view and understanding of the fungal immune-related gene networks remains incomplete, we have gained ground in the pursuit of establishing the molecular basis of fungal immunity, since the introduction of this concept more than a decade ago.

## Fungal allorecognition genes establish evolutionary parallels with eukaryotic and prokaryotic innate immune systems

### Fungal NLRs control allorecognition

An early hint of the relations between fungal allorecognition determinants and *bona fide* immune players from animals and plants, consisted in the finding that several *het* genes encode members of the large STAND protein family (Signal-transducing ATPases with Numerous Domains)^75^ and more specifically NLRs or NLR-like proteins (NOD-like receptors or alternatively NBs-LRR proteins – Nucleotide-binding and Leucine-rich repeat domain).^76^^,^^77^ NLRs are intracellular pattern-recognition receptors (PRRs)^78^ activated by diverse pathogen-associated molecules, danger signals and modified endogenous proteins to mediate innate immunity in animals and plants.^79^^,^^80^^,^^81^ Recently, NLR-like proteins have been shown to mediate prokaryotic anti-viral immunity, recognizing conserved bacteriophage proteins.^82^^,^^83^ NLRs exhibit a typical tripartite domain organization with a central nucleotide–binding and oligomerization (NOD) domain flanked by an N-terminal accessory (or effector) domain and a C-terminal sensor domain, which is formed by superstructure-forming repeats of the canonical LRR-type in plants and animals and predominantly other repeats (i.e. WD40 or tetratricopeptide repeat (TPR)) in bacteria and fungi.^79^^,^^83^^,^^84^ The NOD module, which can be of the NB-ARC^85^ or NACHT^86^ type, plays a critical role in the signaling process of NLRs and mediates the formation of highly-ordered disc-like signalosomes, termed *inflammasomes* in animals^87^ and *resistosomes* in plants.^88^ An anecdotal note that underlines the connection between incompatibility and NLR-function is that the H in the NACHT acronym comes from the *het-E* incompatibility gene from *Podospora*.^86^

Fungal NLRs controlling allorecognition have been recently reviewed in greater detail.^74^ Five out of seven incompatibility systems in the model *Podospora anserina* involve directly or indirectly (in one case) an NLR-encoding gene. Similarly, in the two other model species for fungal allorecognition *C. parasitica* and *Neurospora crassa*, NLRs control cell death to prevent cytoplasmic mixing between strains, with two out of six NLR-dependent systems in *Cryphonectria* and only one in *Neurospora*.^89^ However, in the latter, more than half of the genetically identified allorecognition systems remain to be characterized molecularly. *Neurospora*’s PLP-1 NLR recognizes the SNARE protein SEC-9.^89^ SNAREs (Soluble N-ethylmaleimide-Sensitive-Factor Attachment Protein Receptor) mediate membrane fusion and are essential for vesicle trafficking and exocytosis.^90^ The human homolog of SEC-9, SNAP-25 (Synaptosomal Associated Protein of 25-kDa), regulates exocytosis in neurons, tethering the exocyst complex to the plasma membrane and is the target of the infamous botulinum toxin.^91^^,^^92^^,^^93^^,^^94^ PLP-1 is currently one of the best characterized fungal NLRs and sole for which oligomerization has been supported by experimental evidence.^89^ Oligomerization – one of the key features of NLR signaling – has also been implied for the NWD2 NLR from *P. anserina*.^95^^,^^96^ For PLP-1 and NWD2, as well as two other allorecognition-controlling NLRs from *P. anserina* – the paralogs HET-E and HET-D^97^ – biochemical and genetic evidence point to the C-terminal sensor domains as mediating ligand-recognition and control of the cell death-inducing activity of the receptors.^65^^,^^89^^,^^96^

In almost all characterized cases, NLR-dependent RCD in fungi is induced in response to relatively small endogenous proteins, like SEC-9 or HET-C, which is a 208-amino acid residues protein with a GLTP fold (glycolipid transfer protein).^98^ The *het-c* gene is highly polymorphic with eleven identified *het-c* alleles in a population of 110 *P anserina* strains.^71^ Allelic variants of HET-C are recognized by a subset of allelic variants of HET-E and HET-D.^71^^,^^99^^,^^100^ The *het-e* and *het-d* genes are multiallelic paralogs. Protein variants differ almost exclusively in the WD40 repeats composition of their C-terminal domains, determining allelic specificity (allorecognition-inducing profile in response to variants of HET-C).^96^^,^^97^^,^^101^

**Figure 2:**
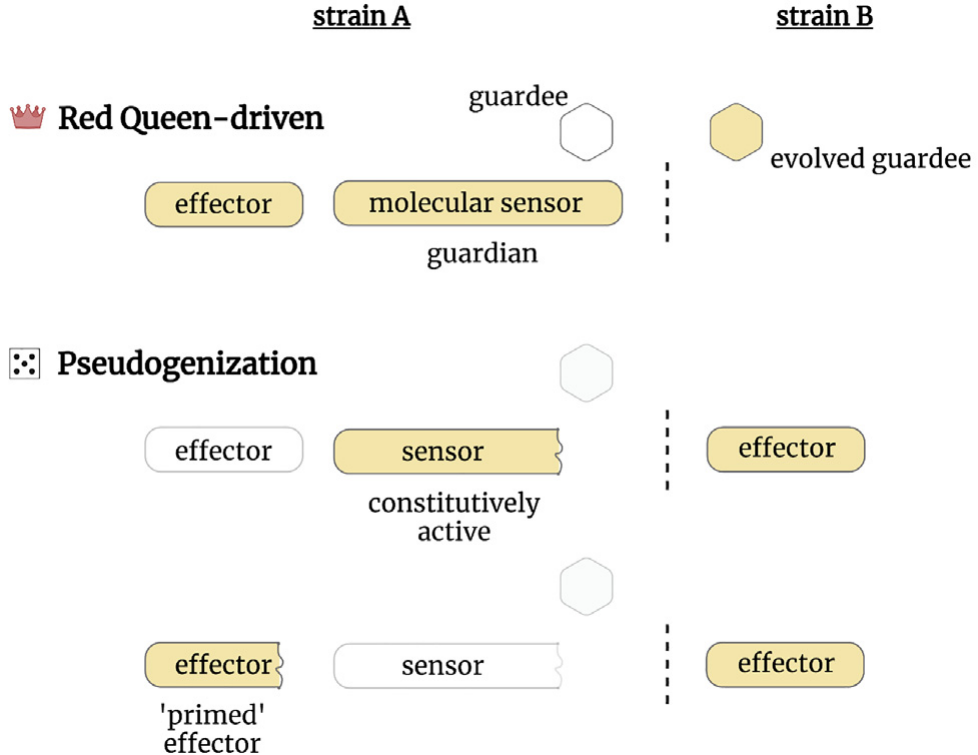
Proposed models for the emergence of RCD-controlling allorecognition systems in fungi Incompatibility systems (between different genotypes/strains) emerge from predating cell death pathways. The incompatibility determinants for all models are highlighted in yellow. In the first proposed model (Red Queen), the arms race-driven diversification of endogenous proteins (guardees), targeted by pathogens, results in inappropriate cross-activation with their respective molecular sensors (guardians) from different strains, after anastomosis (dashed line) (examples: *het-e*/*het-c*, *plp-1*/*sec9*). The cell death effector (executioner) can be a separate downstream protein or a dedicated domain, integral to the sensor. In the second proposed model (pseudogenization), allorecognition systems emerge after a genomic rearrangement leads to a constitutively active sensor, which now can trigger RCD with strains expressing the effector (example: *het-Q1*/*het-Q2*). Alternatively, in some pseudogenization cases, the genomic rearrangements (or mutations) can lead to ‘primed effectors’, which can activate another effector variant (examples: *rcd-1*/*rcd-2* and *het-S*/*het-s*). In such pseudogenization-emerging systems, remaining components of the predating signaling pathways are lost or also subject to pseudogenization (cartoons with increased transparency). The pseudogenization model would represent alternate mechanism to the derivation of allorecognition systems from guardian-guardee pairs, in which concomitant roles in both xeno- and allorecognition is possible.

Single-domain GLTPs bind and transfer sphingolipids between different membrane compartments and are well conserved in eukaryotes.^102^ Intriguingly, human GLTPs, including the ortholog of HET-C, have been implicated in the regulation of autophagy and inflammation,^103^ specifically inflammatory cell death pathways like *necroptosis*.^104^ While another HET-C ortholog, named ACD11 (Accelerated Cell Death 11) from *Arabidopsis thaliana,* controls necrotic immune-related cell death reaction termed *hypersensitive response* (HR).^105^^,^^106^ The deletion of the *acd11* gene induces HR in the absence of any pathogens. Plants can be rescued from the autoimmune cell death reaction by mutations in a gene encoding an NLR protein named LAZ5 (Lazarus 5).^107^ The functional association between LAZ5/ACD11 has found a potential explanation within the ‘guard hypothesis’,^74^^,^^108^ which stipulates that some NLRs surveil – or ‘guard’ – the state or integrity of key endogenous proteins (‘guardees’), targeted by the virulence factors of pathogens. Noteworthy, a recent report documents a virulence factor from the oomycete *Phytophthora capsici* targeting binding-partners of ACD11.^109^ Remarkably, the exocyst complex has also been identified as a frequent target of pathogens in animals^110^ and plants.^111^^,^^112^^,^^113^^,^^114^ Overall, these observations and the ‘guard hypothesis’ provide a plausible explanation for the recurrent emergence of NLR-based allorecognition systems in fungi (Figure 2). Arms race-driven evolution of the guardees and the guardians generates a variety of different alleles, which can lead to an inappropriate activation of RCD, in absence of a pathogen, when co-expressed in the same cells. A robust functional link between some fungal NLRs and their guardees, likely because of the importance it carries outside of allorecognition, is further suggested by the discovery that an NLR/SEC-9 incompatibility system has emerged independently in four distant fungal species.^89^^,^^115^ Yet, the biological roles outside of allorecognition and the precise mechanism of action for such hypothetical guardian-guardees pairs remain to be established.

### Allorecognition determinants carry TIR-related domains

The *trans*-kingdom evolutionary parallels extend further when we focus on the N-terminal effector/signaling domains of NLRs. For example, genome-mining has identified a small group of fungal NLRs with an N-terminal TIR (Toll-interleukin-1 Receptor) domain.^74^ TIR domains play a central role in immunity in animals, plants and bacteria.^116^ TIR domains consist of ∼150–200 amino acid residues, mediate homotypic protein-protein interactions in the formation of large signaling complexes.^117^ In addition, TIR domains were recently uncovered as NAD^+^-consuming enzymes (NAD^+^ hydrolase) and some can also hydrolyze dsDNA/dsRNA,^118^^,^^119^^,^^120^^,^^121^ producing cyclic nucleotides-based immune signaling molecules, some of which represent derivatives of cADPR (cyclic ADP-ribose).^122^^,^^123^^,^^124^^,^^125^ The allorecognition-controlling HET-E, HET-D and HET-R NLRs, whereas not carrying a *bona fide* TIR domain, exhibit an N-terminal HET domain, which has been found to share remote sequence homology with TIR.^84^ The HET domain is sufficient to induce cell death in fungi and has been frequently identified on allorecognition determinants.^37^^,^^45^ In *N. crassa*, four out of six studied incompatibility systems rely on a protein with a HET domain (*tol*, *pin-c*, *het-6*, *het-e* genes), none of which belongs to the NLR family. The situation is similar in *C. parasitica*, with half of the identified six systems involving a HET domain (*vic1*, *vic6* and *vic7* genes).^126^^,^^127^ Better molecular and structural characterization of the HET domain is needed to decipher its function and compare with TIR domains. Yet, the sequence homology, predicted structural models and protein architecture positioning (both TIR and HET being N-terminal NLR domains) would suggest that this landmark domain of fungal allorecognition is a distant relative of the xenorecognition and immune cell death-controlling TIRs. The establishment of the enzymatic activity of the HET domain would further broaden the role of fungal NADases, which recently have been proposed to play a role in biotic interactions.^128^

### Allorecognition systems relate evolutionary to necroptosis and pyroptosis

The exploration of fungal allorecognition has also established multiple evolutionary links between fungal RCD pathways and the metazoan cell death programs *necroptosis* and *pyroptosis*. These are lytic cell death reactions in response to intracellular pathogens or danger-associated molecular patterns, resulting in the release of pro-inflammatory cytokines and other immunogenic cellular content.^129^ Necroptotic and pyroptotic cell death rely on executioner proteins targeting the cellular membrane. In necroptosis, the cell death executioner is the MLKL protein (Mixed-lineage Kinase Domain-like), which acts downstream of RIPK3 (Receptor-interacting Protein Kinase 3).^130^^,^^131^ The latter phosphorylates MLKL directly when recruited in an amyloid-mediated protein complex with RIPK1, termed the necrosome, which activates the cell death executioner.^132^^,^^133^ Phosphorylated MLKL oligomerizes in the plasma membrane, where an N-terminal helix of its 4HB (Four-Helix bundle) domain plays a key role in membrane permeabilization and cell death induction.^134^^,^^135^^,^^136^ The ‘killer’ 4HB domain of MLKL has a fungal homolog, named HeLo or HeLo-like (HELL), which can be found as an N-terminal domain of some fungal NLRs or on cell death effectors controlled by fungal NLRs.^137^ In addition, a HeLo/4HB homolog has also been identified in plants, as an N-terminal domain of a sub-class of NLRs, notably CNLs (Coil-coiled NLRs).^137^^,^^138^

The best characterized HeLo-containing cell death effector and allorecognition determinant is *P. anserina* HET-S protein.^139^ The HET-S protein consist of two domains – the cytotoxic HeLo domain at its N-terminus and a C-terminal amyloid, prion-forming domain (PFD).^140^^,^^141^ Amyloids are fibrillar, highly cooperative β-sheet rich protein folds.^142^ The pore-forming protein can be activated by a naturally occurring HeLo-dead mutant variant of itself, termed HET-s (small s). HET-s differs from HET-S (large S) at only 14 amino acid positions and a single substitution (H33P) situated in the transmembrane N-terminal helix of HET-S HeLo domain is sufficient to explain the allelic specificity of the two variants.^143^ Deprived of cytotoxic properties, HET-s behaves as an infectious protein or a prion, spontaneously assembling into orderly self-propagating amyloid fibers, constituted by the prion-forming domain.^144^^,^^145^^,^^146^ These amyloids serve as a structural template for the PFD of HET-S during the allorecognition cell death reaction.^147^ The transconformation of HET-S PFD from an unstructured state toward the amyloid fold acts as a trigger for the HeLo domain and leads to the release of an N-terminal transmembrane α-helix, which attacks the plasma membrane, similarly to MLKL 4HB.^147^^,^^148^^,^^149^^,^^150^

Beside its role in the *het-S*/*het-s* allorecognition system, HET-S can be activated by the NLR protein NWD2 encoded by the adjacent gene of *het-S* in the genome of *P. anserina*.^95^^,^^96^ The N-terminal effector domain of NWD2 is a short amyloidogenic motif, showing sequence homology to the PFD of HET-S, and capable of adopting a HET-S-like amyloid fold. In the current model, formation of an NWD2 signalosome leads to the cooperative folding of the amyloid fold, which then templates the PFD of HET-S, triggering the activity of the cytotoxic HeLo domain.^96^ The signal-transducing mechanism thus consists of a structural templating form the receptor toward the downstream effector and executioner protein. The *het-S*/*nwd2* two-gene cluster is conserved in various fungal genomes, while the allorecognition system *het-S*/*het-s* is exclusive to *P. anserina*.^151^ Moreover, NLR-dependent amyloid-based signaling has been found to rely on a large variety of amyloidogenic sequences.^152^ One of the fungal signaling amyloids – termed PP (pseudo-palindromic) – appears homologous to the amyloid domain mediating the formation of the RIPK1/RIPK3 necrosome, termed RHIM (RIP homotypic interacting motif).^132^^,^^137^^,^^153^ An evolutionary link to RHIM has previously been proposed for the PFD of HET-S.^154^ These findings further underscore the relation between amyloid-based NLR signaling in fungi and mammalian necroptosis. Intriguingly, the discovery of signaling amyloids in bacteria has recently extended the evolutionary link to prokaryotes.^155^

The evolutionary relation between *pyroptosis* and fungal cell death pathways has been established recently, notably with the discovery and characterization of fungal gasdermin-like proteins controlling allorecognition in *P. anserina* and *N. crassa*.^156^^,^^157^ Gasdermins are a family of pore-forming membrane-targeting proteins, executioners of pyroptotic cell death in mammals and of anti-phage immune cell death in bacteria.^158^^,^^159^^,^^160^ In animals and bacteria, characterized gasdermins are generally activated through proteolytic cleavage by inflammatory caspases and other proteases, which remove the inhibitory C-terminal domain of the gasdermin freeing the pore-forming domain.^161^^,^^162^ Similar proteolytic cleavage has been reported for the fungal gasdermin HET-Q1 by the serine protease HET-Q2 during the allorecognition cell death reaction in *P. anserina*.^163^ The second allorecognition-associated gasdermin, RCD-1 (Regulator of Cell death) from *N. crassa,* appears as an exception in regards to its activation, which depends on the co-expression of two distinct antagonistic allelic variants of the protein.^156^ Currently the mechanism of cross-activation between the two RCD-1 variants is unknown. Fungal gasdermins (fGSDMs), bacterial gasdermins (bGSDMs) and mammalian gasdermins exhibit sequence and structural homology exclusively over the pore-forming N-terminal domain.^164^^,^^165^ A notable difference between mammalian and microbial gasdermins resides in the inhibitory C-terminal domain, which is significantly shorter in the latter group and the reported structures of two bGSDMs unveil unsurprisingly a different mechanism of inhibition.^160^ Precise structural information regarding fGSDMs and their pores is yet to be reported, which should allow for a fascinating *trans*-kingdom comparison of these ancient pore-forming proteins.

### Allorecognition cell death: A gateway to the fungal immune system

**Figure 3:**
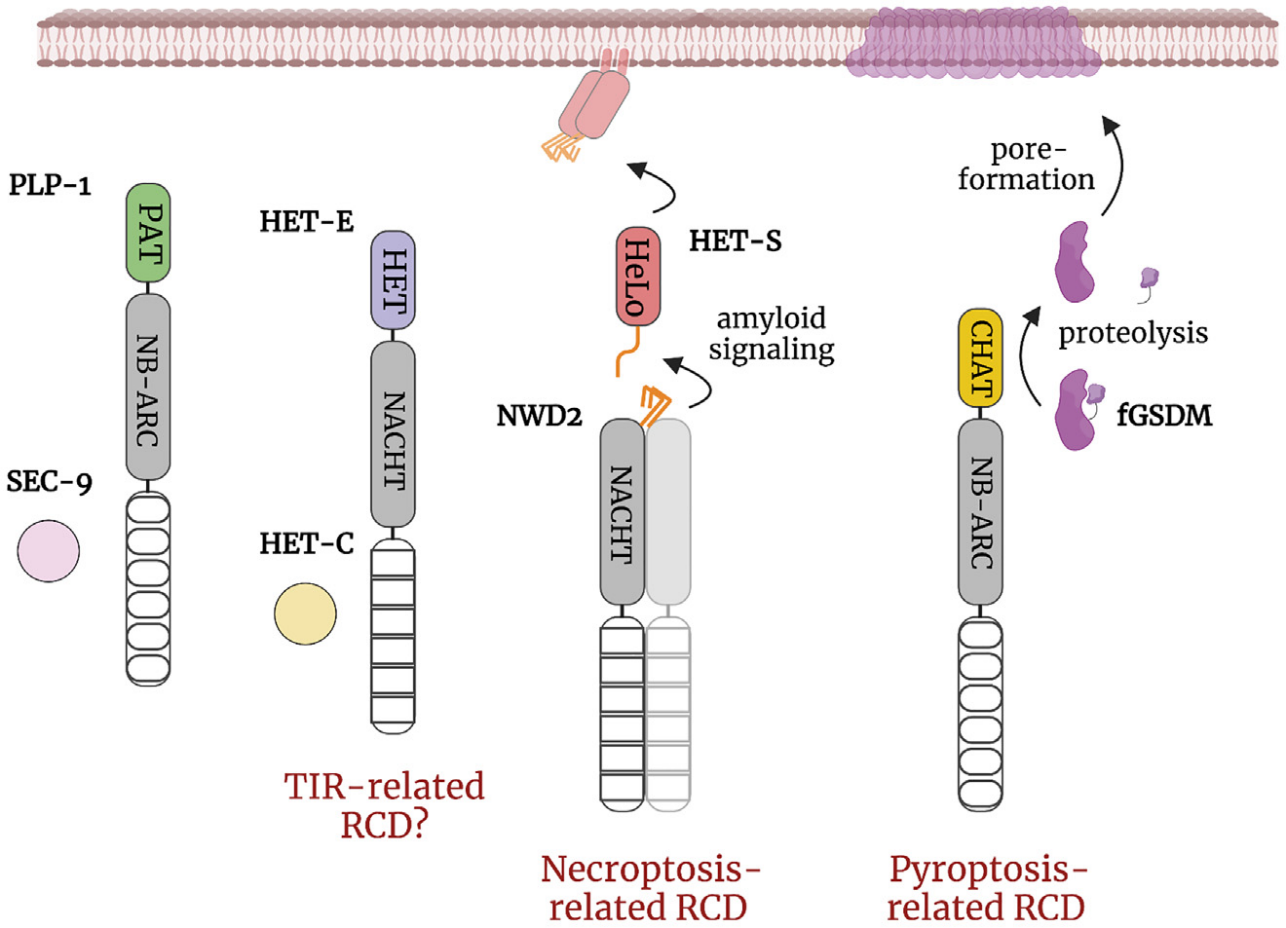
Cell death pathways controlling fungal allorecognition show evolutionary ties with eukaryotic immune systems The cartoon summarizes the key evolutionary parallels that have emerged from the molecular characterization of fungal incompatibility systems. The NLR protein family takes central stage in fungal immunity. Shown are selected NLR-dependent allorecognition systems – PLP-1/SEC-9 from *Neurospora crassa* and HET-E/HET-C from *Podospora anserina* – and NLRs involved in RCD pathways, evolutionary related to mammalian necroptosis and pyroptosis. The N-terminal effector/signaling domains are highlighted in different colors: patatin-like phospholipase (PAT) in green, HET/TIR-like in purple, the HeLo domain (red), distantly related to MLKL 4HB (necroptosis executioner protein) and RPW8-like coiled-coil domains from plant NLRs, controlling cell death in the hypersensitive response. The NWD2 NLR controls the HET-S pore-forming protein through amyloid-based signaling (orange domain). Some fungal signaling amyloids are related to necroptosis-controlling RHIM amyloid motif. Shown is a contracted fungal pyroptotic-like cell death pathway. The caspase-related CHAT domain (yellow) activates the fungal gasdermin (fGSDM) by proteolysis of its inhibitory C-terminal domain. fGSDM then oligomerizes in the plasma membrane to induce cell death. Hypothesized aspects of these models are shown with increased transparency.

To summarize, the exploration of regulated cell death in the context of fungal allorecognition has systematically uncovered molecular determinants evolutionary related to immune genes outside of the fungal kingdom (Figure 3). The most recent findings – linking fungal cell death to pyroptosis and necroptosis – support the Paoletti-Saupe hypothesis that fungal allorecognition systems originate from predating gene networks with roles outside of the allorecognition phenomenon and likely dedicated to heterospecific non-self discrimination. Below, I will describe more in depth the gene families, acting as gene pools, for the recurrent emergence of allorecognition systems.

## Submerged side of the iceberg: Gene repertoires and networks of the fungal immune system

The vast majority of fungal allorecognition genes belong to large and widespread gene families. The three main gene families, serving as reservoir for allorecognition systems, appear to be the HET/TIR domain-encoding genes, the gasdermin and NLR gene families. The biological roles of these gene families remain to be uncovered. Yet, the ever-growing number of sequenced fungal genomes offers the possibility to compile extensive gene catalogs and gain insight into their distribution and evolutionary regimes.

### NLRs in fungi: Abundant and diverse

Genome-mining approaches have been twice applied to identify and list fungal NRLs.^74^^,^^84^ The initial report by Dyrka et al., uncovering the broad distribution and diversity of NLR-encoding genes in fungi, has been recently updated with nearly four times more genomes (882 strains, 561 species).^74^ NLR-encoding genes were present in the genomes of the vast majority of filamentous species, both ascomycetes and basidiomycetes, and absent in yeast genomes. The latest analysis confirms the great inter-species variability of NLR gene content in fungi, establishing a median of 41 NLRs and a mean of 57 NLRs per genome with at least one hit. Basidiomycete species contained on average ∼60% more NLRs (mean of 83 per genome) in comparison to ascomycetes (mean of 52 per genome). Dozens of species carry NLR repertoires consisting of hundreds of genes, reaching a record of 602 NLRs in the basidiomycete *Fibularhizoctonia*. The genomes of the two allorecognition model species *P. anserina* and *N. crassa* contain 77 and 17 NLR genes, respectively.^84^ The highly variable numbers of NLR genes in different fungal species reflects the distribution of this gene family in plants and animals.^79^ Moreover, strain-specific gene expansions (high number of paralogs) and frequent inter-genic recombinations, leading to a rapid decrease of established NLR gene orthologies, indicate a *birth-and-death* evolutionary mode. Such heightened evolutionary dynamics could be explained as the likely cause and consequence of niche-specific adaptations to an ever-changing landscape of biotic and exogenous non-self cues.

**Figure 4:**
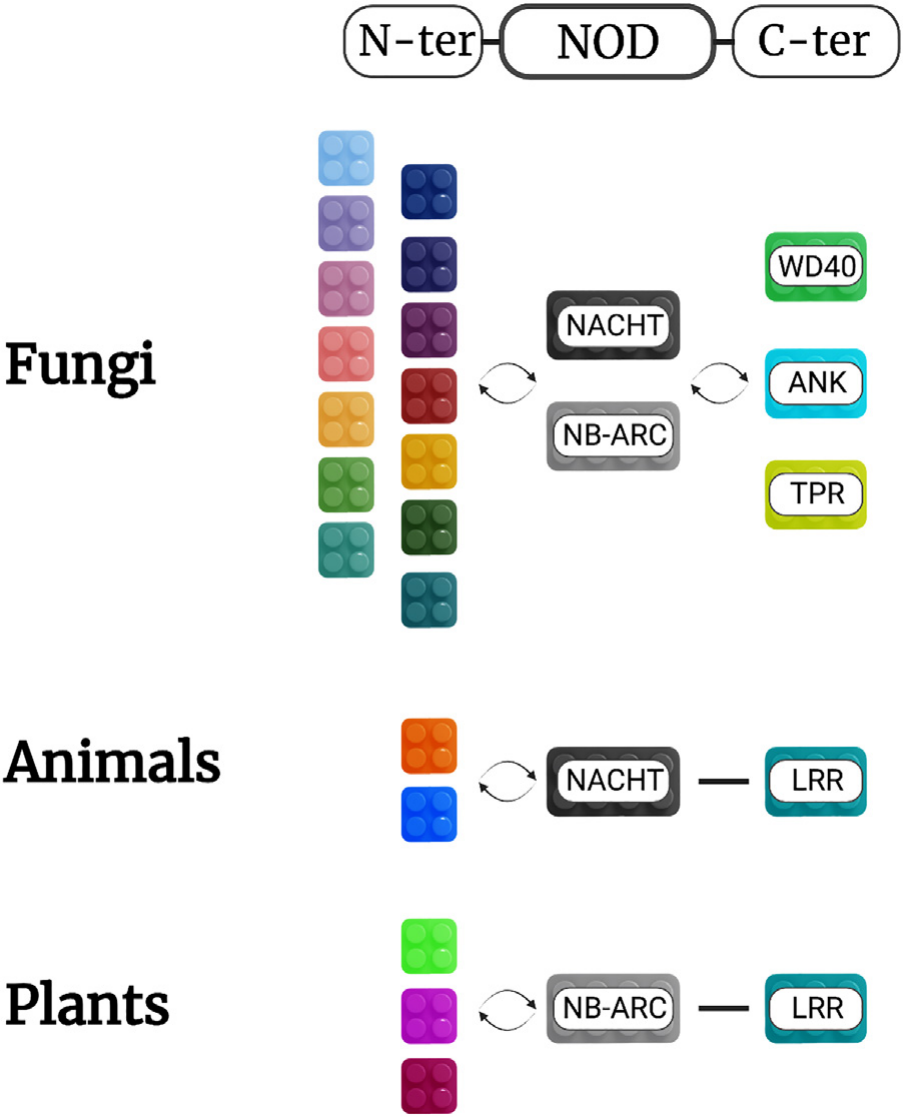
A diagram of the architectural diversity of NOD-like receptor (NLRs) proteins in fungi, animals and plants Colored blocks represent protein domains situated at the N-terminus, centrally or the C-terminus of NLRs. The cartoon underscores the striking diversity of fungal NLRs with currently 14 different N-terminal modules, representing different domain families (Wojciechowski et al.). This is in stark contrast to the limited number of effector/signaling modules found in animal and plant NLRs: TIR, CC and CC_RPW8_ in plants, CARD and PYD domains in animals.

NLRs are modular proteins.^79^ The different types of domains composing each of the three canonical ‘modules’ (N-terminal effector domain, central NOD and C-terminal sensor domain) can be combinatorially assembled into a variety of protein architectures (Figure 4). One of the most striking features of the fungal NLRs is the significant diversity of N-terminal effector/signaling domains^166^ and C-terminal superstructure-forming repeats that constitute the various members of the family.^84^ This contrasts with plant and animal NLRs, which show much lower diversity of NOD-associated domains (Figure 4). From an analyzed set of more than 36000 fungal NLR proteins, more than fourteen different effector/signaling domains have been annotated and grouped into separate clades.^74^^,^^166^ The majority of the N-terminal annotations (10 out of 14 clades) have been predicted to carry an enzymatic activity. These enzymatic effector/signaling domains fall into four main categories: hydrolases, phosphorylases, kinases, and peptidases. Among the hydrolases, we find the TIR/HET clade (discussed previously), the SES-B-like (SBL) α/β-hydrolases clade and the patatin-like phospholipases. The latter have been shown to hydrolyze membrane lipids and control immune-related cell death in plants.^167^^,^^168^^,^^169^ A patatin-like phospholipase domain, situated on the PLP-1 NLR protein controlling allorecognition in several fungal species, has also been determined as essential for the induction of RCD.^89^ Another frequent N-terminal signaling domain is the PNP_UDP hydrolase (PF01048),^74^ which has recently been described in bacteria as an immune-related ATP nucleosidase.^170^ Next, the RelA/SpoT hydrolases, which represent a minor yet intriguing clade of NLR effector domains with RelA/SpoT homologs (RSH) mostly characterized in bacteria. RSH control the production of the alarmones guanosine penta- and tetraphosphate – (p)ppGpp – and regulate stress-induced metabolic adaptations termed the ‘stringent response’.^171^^,^^172^ The role of these two metabolites in fungi is poorly documented, although their presence has been detected in yeast.^173^ A particularly interesting study, considering the hypothesis that fungal NLRs mediate xenorecognition, indicates that the stringent response and (p)ppGpp repress the antifungal activity of *Pseudomonas* strains, used otherwise as a biocontrol agent.^174^

Another category of enzymatic effector domains found in fungal NLRs, contained the peptidases with two main clades: the serine S8 peptidase family and the cysteine peptidases of the CHAT family. CHAT (Caspase Het-F associated with TPRs) peptidases are closely related to the caspase family.^175^ Noteworthy here is a recent finding that CHAT proteases control some fungal gasdermins^163^ and are integral to gasdermin-dependent anti-phage systems in bacteria.^165^ Despite the high diversity of enzymatic effector domains, more than half of the annotated N-terminal sequences belonged to two clades – Goodbye-like (GBL) and HeLo-like (HELL) – related to the 4HB domain of the necroptosis executioner protein MLKL and coil-coiled (CC) domains found on plant NLRs.^166^ The activity of HeLo/HELL effector domains relies on an N-terminally-situated α-helix with transmembrane propensity, representing a key functional element during membrane targeting.^137^^,^^147^ The precise mechanism of cell death remains to be elucidate, however, work on MLKL^176^ and the CC-domain-containing ZAR1 NLR from plants^177^ suggests that the GBL/HELL clades of fungal NLR effector domains might form cation channels, triggering cell death. Overall, the analysis of the effector/signaling NLR domains in fungi indicates that these receptors control a variety of signaling pathways with diverse downstream responses.

A crucial open question, essential to uncover the biological roles of fungal NLRs, relates to the diversity of their C-terminal sensor module and in particular to the unidentified molecular cues activating the receptors. While looking for answers, comparative genomics analyses unveil the extreme diversity of the C-terminal NLR domains, most often composed of ANK, TPR or WD40 repeats.^84^^,^^178^ The sequences of such repeats vary in length between 20 and 40 amino acids and their folds consist of 3–4 short secondary structure elements. The sensing domain is thus generally composed of a pattern of subsequent individual repeats. Several reports have documented the role in recognition and sensing of the C-terminal module of fungal NLRs, also uncovering amino acid residues under positive diversifying selection, predicted to sit on the solvent-exposed surfaces of the sensing domains.^65^^,^^89^^,^^101^ Such evolutionary marks could be explained by the continuous need for adaptation to change and to keep track with fast evolving molecular cues, potentially in arms race with other organisms. Other mechanisms have been described that accelerate the genesis of this recognition repertoire, notably the concerted evolution of a subset of repeats with very high internal sequence conservation. The highly conserved repeats can be constantly shuffled through genetic recombinations inside the same gene or in-between genes.^101^ The combinatorial rearrangements result potentially in a very high number of distinct sensory C-terminal domains, which at species level might represent an extremely diversified NLR repertoir.^65^ Inter-allelic recombination has previously been proposed as a diversification mode for plant NLRs^179^^,^^180^ and unusual exon shuffling processes have also been evidenced in NB-ARC-TPR architecture genes in brown algae.^181^ Alternatively, in fungal species like *Tuber melanosporum*, sensor NLR domains can be diversified post-transcriptionally, through alternative splicing of 3-bp long microexons.^182^ These findings underscore the need for constant diversification of the sensing domains of fungal NLRs and strongly suggest that the molecular receptors follow highly diverse molecular cues.

The variability of NLR architectures in fungi, achieved through the combinatorial rearrangements of N- and C-terminal modules, has recently been expanded with the reports of a group of fungal NLRs carrying two distinct consecutive N-terminal effector/signaling domains.^166^ This class of receptors are reminiscent of human NAIPs (NLR family apoptosis inhibitory proteins), which carry three BIR domain repeats at their N-termini.^183^ However, in fungi, the two-effector domains of such NLRs exhibit distinct putative functions – a protease and a membrane targeting domain, for example –, suggesting a direct multifaceted outcome of the receptor activation and a likely synergy between some effector/signaling domains during the response. Synergetic NLR-dependent RCD signaling has been previously proposed for two different amyloid-controlled effector proteins in *Chaetomium globosum*.^137^ For both, the ‘all-in-one’ architectures (with a single or a double effector domain) and the amyloid-dependent NLR signaling, there are still many intriguing mechanistic details remaining to be explored and described. This would result not only in a better understanding of the cell death pathways in fungi but also in a meaningful contribution toward NLR biology.

### HET domain-encoding genes: Mysterious players of the fungal immune system?

The HET domain, while described as a signaling module on fungal NLRs, appears often as the sole identifiable domain encoded by other genes, amounting to more than a hundred in some species (i.e., *P. anserina,* 125 loci).^45^^,^^184^ The HET domain is not only one of the most abundant domains in fungal genomes but also one that shows a high copy number inter-species variance. In fungal comparative genomics studies, HET domain genes pop up as highly variable gene family with lineage or species-specific expansions.^185^^,^^186^^,^^187^^,^^188^^,^^189^^,^^190^ The observed high numbers of HET domain-encoding genes in a given species are often interpreted, likely mistakenly, as the result of a specific requirement for incompatibility systems. However, this interpretation neglects that only a minute fraction of HET domain-encoding genes function in allorecognition (a ratio of 5:125 genes in *P. anserina*).^37^ The genomic distribution of HET domain-encoding genes, combined with the reported evolutionary relation between HET and TIR domains,^84^ lead to the hypothesis that HET domain-encoding genes might play an important role in fungal immunity.

Despite being abundant and with broad phylogenetic distribution, no recent evolutionary analysis has been performed on fungal HET domain proteins. Nevertheless, Zhao et al. had provided exhaustive species-level analyses of HET domain-encoding genes, using *N. crassa*.^39^ The comparative genomics approach uncovered 73 HET domain-encoding loci in 25 sequenced wild isolates of *N. crassa* and 69 genes in the reference *N. crassa* genome. Four HET domain-encoding genes were identified as strain-specific in *N. crassa,* and 69 genes presented true orthologs in the closely related *Neurospora tetrasperma* species. Otherwise, HET domain-encoding genes, similarly to the NLR genes, appeared poorly conserved between more distant species.^39^ In accordance with the latter observation, Zhao et al. report that the HET domain-encoding genes in *Neurospora* are often hypervariable with 34 out of the 73 identified loci showing high sequence variability and multiallelism. Signs of balancing selection – long-diverged haplogroups – were uncovered for 15 such hypervariable HET domain-encoding genes. This comparative genomics approach has been used by the authors to identify a novel allorecognition gene (termed *het-e*) in *N. crassa*, however what are the driving forces diversifying the HET domain genes, remains an open question, similarly to the precise molecular mechanisms of HET domain-mediated cell death. To investigate the latter, it would be necessary to first demonstrate the TIR-like activity of fungal HET domains before characterizing the functions of the unannotated protein sequences often spanning the HET domain. The high number of HET domain proteins per species combined with the lack of clearly assigned protein architectures should incite us to see this important protein family as one of the most obvious knowledge gaps to address in the pursuit to establish the basis of fungal immunity.

### fGSDMs and their proteases: Two-gene clusters controlling RCD

**Figure 5:**
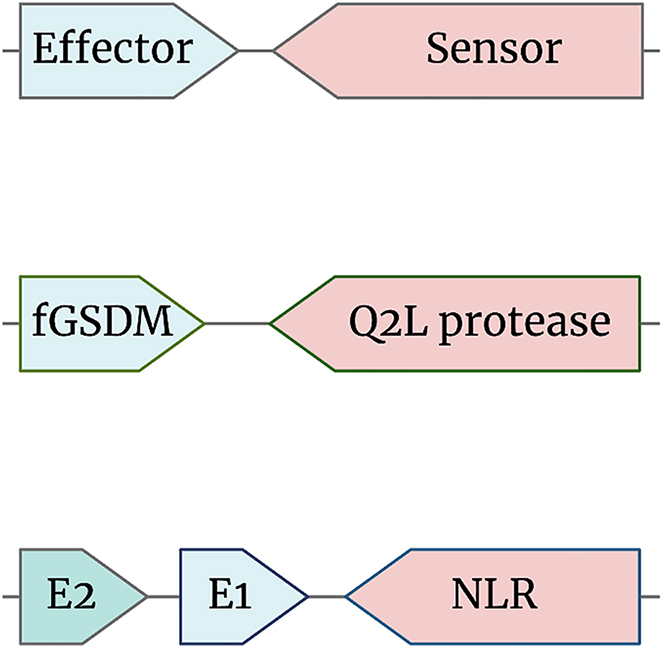
Micro-clustering of RCD pathways in fungi

The recently characterized cell death executioners – the fungal gasdermins RCD-1 (*N. crassa*)^156^^,^^157^ and HET-Q1 (*P. anserina*)^163^ – also belong to a widespread in fungi and relatively abundant gene family. Genome mining has identified ∼1900 fGSDMs in 400 fungal genomes with dozens of species carrying >10 genes and some >20 genes.^157^ fGSDM genes appeared clearly more abundant in Ascomycota and like the NLR family, showed signs of death-and-birth evolutionary regime with scattered gene phylogeny and strain-specific gene expansions. The molecular characterization of some fGSDMs indicates that these share key characteristics (i.e. membrane targeting, oligomerization) with gasdermins in animals and bacteria, including their proteolytic mode of regulation. Regarding this latter point, a somewhat surprising finding revealed that ∼80% of fGSDM genes are genomically clustered with the genes encoding their respective protease-bearing upstream activators. The majority of fGSDMs (∼63%) were clustered with genes encoding serine S8 proteases and another 19% were in vicinity (within 10 kb) to genes encoding caspase-related CHAT proteases (Figure 5). Similar genomic organization of gasdermin and protease-encoding genes has been uncovered in bacteria, where the gasdermin-dependent cell death reaction prevents the spread of bacteriophages.^160^ Thus in microbes (fungi and bacteria), the two-gene clusters represent functional units, where one gene encodes a pore-forming protein and the other a molecular sensor that controls its cytotoxic activity.

The bioinformatics analysis of the fGSDM-controlling proteases – named Q2L (after the HET-Q2-like serine S8 protease discovered in *P. anserina*) – has unveiled a great diversity of protein architectures among these receptors.^163^ Q2L proteins appeared often multidomain with 18 different protein architectures been identified, comprising roughly a third of the retrieved ∼1500 protein sequences. In most of the annotated sequences, next to the putative protease domain, were identified regions consisting of superstructure-forming repeats, belonging to one of the three classes (ANK, TRP and WD40) frequently associated with fungal NLRs. Moreover, a subset of sequences was identified as belonging to the NLR protein family. Among these sequences were dozens of NLRs or NLR-like proteins carrying a caspase-related CHAT protease. Thus, some of the two-gene clusters appear to encode for contracted pyroptotic-like pathways, where the caspase is physically part of the receptor, whereas in mammals the NLR receptor controls a separate caspase protein directly or through an adaptor protein.^191^^,^^192^ The variability of annotated Q2L architectures indicates that fGSDMs are integral to diverse signaling pathways and suggests multiple different regulatory modes of gasdermin cell death in fungi.

The discovery of genomic clustering between gasdermin and protease-encoding genes increased the number of cell death-controlling gene clusters in fungi, suggesting that such genomic arrangements of genes associated with non-self discrimination and organismal defense might be more frequent than anticipated. Yet, this finding is less surprising when one considers the reports of metabolic gene clusters (MGCs) in fungi.^193^ MGCs consist of two or more genes encoding for discrete metabolic pathways that determine the biosynthesis or degradation of primary and secondary metabolites with a variety of roles, one of which is chemical defense and the production of toxins against antagonistic microbes, insects and predators.^22^ Defense genes are also often clustered in bacterial genomic regions termed ‘defense islands’, collectively representing a diverse and rich repertoir of anti-phage systems.^194^^,^^195^ Considering the description of fungal MGCs and the characterized two-gene immune-related clusters, one could hypothesize that some genomic regions in fungi might be analogous to bacterial defense islands. The non-random genomic distribution of HET domain-encoding genes in several different fungal species^39^ would suggest that such a hypothesis seems plausible. Broader analysis with the entirety of presently identified immune-related gene families would be necessary to evidence the potential genomic clustering of these genes and establish a clear analogy with bacterial defense islands. Hypothetically, the existence of such genomic regions in fungi could equally be used to uncover additional defense systems, inferring functionality by the genomic context.^196^ The use of a similar approach has been fruitful in expanding the repertoire of bacterial immune-related genes.^197^

In addition, the combinatorial nature of already described RCD-associated domains could be used in genome mining analyses to search for novel molecular players with putative roles in organismal defense and management of biotic interactions. Extra efforts should be directed into gaining a fuller image of the gene networks defining biological individuality in fungi and explore the extent to which the three currently characterized, and partially intertwining, gene families (NLRs, fGSDMs and HET domain genes) constitute the core of the fungal immune-related RCD repertoires. A hint to the central place they take among immune-related genes is further provided by the recent molecular characterization of *Aspergillus fumigatus* allorecognition determinants, the majority of which fell into some of the three gene families or carried NLR-associated effector domains.^198^

## Fungal immunity beyond host defense

Although the general contexts of allorecognition and cell death have naturally directed our approach to fungal immunity toward host defense, it would be timely to consider the implications of a fully fledged fungal immune system in the broader immunological context of biotic interactions and the limits of biological individuality.^2^^,^^199^ Fungi are found in most habitats on Earth, exhibiting extremely diverse lifestyles.^200^^,^^201^ Fungal species display complex behaviors and have been recently proposed to play a mediating ecological role between organisms from different kingdoms.^202^^,^^203^ The identified fungal RCD-controlling gene families could thus be involved in other roles than host defense like the maintenance of organismal homeostasis or defining the outcomes of a broad variety of fungal biotic interactions, including symbiosis. Mammalian NLRs, for example, have been shown to play a crucial role in the mediation of intestinal homeostasis and the sensing of commensal gut microbiota.^204^^,^^205^ Plant immunity networks also play an important role in symbiotic relations with bacteria and fungi.^206^^,^^207^^,^^208^ Looking for analogies in fungi might seem premature, nevertheless the multiplicity of ecological roles of the putative fungal immune system should certainly be considered.

In this context, the finding that some of the species with highest number of NLR genes in their genomes have been associated with mycorrhizal or endophytic lifestyles, both of which are ecologically important forms of plant-fungal symbiosis,^209^^,^^210^ appears intriguing. More than half (∼70%) of the twenty fungal species with highest number of NLR genes were identified as mycorrhizal or endophytic,^74^ with a median of ∼250 genes per genome. Lineage-specific NLR gene expansions have been previously noted for some mycorrhizal species like *Piriformospora indica*^211^ (272 NLR genes, 25 Mb genome) and *Laccaria bicolor*.^212^ Remarkably, in the latter, NLR-encoding genes were found to be overexpressed during the establishment of the symbiotic relation and also during fruiting body development.^212^ Genes encoding NLRs, superstructure-forming repeats proteins, TIR domain proteins or patatin domain-containing proteins have been found among the differentially expressed genes during mycorrhizal formation in the arbuscular mycorrhizal species *Gigaspora margarita* and ectomycorrhizal species of the *Suillus* genus.^24^^,^^213^ Moreover, in *G. margarita*, the gene expression levels of some of these genes appeared to respond to the presence/absence of endobacteria, which represent the natural microbiota of the fungus.^24^^,^^214^ The endobacteria have been shown to increase the fitness of some mycorrhizal fungi and have been found to inhabit ascomycetes and basidiomycetes.^215^^,^^216^ The relations between the fungi and their microbiota can be close. In some cases, endobacteria have been found to harbor genes from their fungal hosts; strikingly, often these genes have been identified as carrying a HET domain.^217^^,^^218^ Overall, these findings support the idea that fungal immune-related gene networks are involved in the management of diverse biotic interactions.

## Conclusion

In summary, the findings in the past decade have strongly supported the hypothesis that fungi have a fully-fledged immune system. Key discoveries have been made by the study of a lifestyle-dedicated defense mechanism (allorecognition), which however, I argue, is only the most visible side of the molecular networks dedicated to non-self discrimination. The uncovered evolutionary parallels between fungal cell death pathways and immune systems in other taxa have already situated the paradigm of fungal immunity in the field of comparative immunology. Looking into the future, we should be emboldened to tackle some of the remaining major questions like what are the precise roles (mechanisms of action etc.) of the gene networks, from which allorecognition determinants emerge. Answers certainly lie at the complex to study, especially in laboratory conditions, inter-species interactions surface. The increased consideration of fungi as potential hosts and ecological inter-species mediators – and a concerted focus on the immunity-associated gene networks, including using novel experimental approaches – might fill the existing knowledge gaps, contributing significantly toward the assertion of fungal immunity and unveiling an important missing component of the forces shaping ecology.
